# A Practical Do-It-Yourself Recruitment Framework for Concurrent eHealth Clinical Trials: Simple Architecture (Part 1)

**DOI:** 10.2196/11049

**Published:** 2018-11-01

**Authors:** Hannah L Palac, Nameyeh Alam, Susan M Kaiser, Jody D Ciolino, Emily G Lattie, David C Mohr

**Affiliations:** 1 AbbVie Inc North Chicago, IL United States; 2 Center for Behavioral Intervention Technologies Department of Preventive Medicine Northwestern University Feinberg School of Medicine Chicago, IL United States; 3 Division of Biostatistics Department of Preventive Medicine Northwestern University Feinberg School of Medicine Chicago, IL United States; 4 Center for Behavioral Intervention Technologies Department of Medical Social Sciences Northwestern University Feinberg School of Medicine Chicago, IL United States

**Keywords:** eHealth, mHealth, online recruitment, REDCap, referral management

## Abstract

**Background:**

The ability to identify, screen, and enroll potential research participants in an efficient and timely manner is crucial to the success of clinical trials. In the age of the internet, researchers can be confronted with large numbers of people contacting the program, overwhelming study staff and frustrating potential participants.

**Objective:**

This paper describes a “do-it-yourself” recruitment support framework (DIY-RSF) that uses tools readily available in many academic research settings to support remote participant recruitment, prescreening, enrollment, and management across multiple concurrent eHealth clinical trials.

**Methods:**

This work was conducted in an academic research center focused on developing and evaluating behavioral intervention technologies. A needs assessment consisting of unstructured individual and group interviews was conducted to identify barriers to recruitment and important features for the new system.

**Results:**

We describe a practical and adaptable recruitment management architecture that used readily available software, such as REDCap (Research Electronic Data Capture) and standard statistical software (eg, SAS, R), to create an automated recruitment framework that supported prescreening potential participants, consent to join a research registry, triaging for management of multiple trials, capture of eligibility information for each phase of a recruitment pipeline, and staff management tools including monitoring of participant flow and task assignment/reassignment features. The DIY-RSF was launched in July 2015. As of July 2017, the DIY-RSF has supported the successful recruitment efforts for eight trials, producing 14,557 participant records in the referral tracking database and 5337 participants in the center research registry. The DIY-RSF has allowed for more efficient use of staff time and more rapid processing of potential applicants.

**Conclusions:**

Using tools already supported at many academic institutions, we describe the architecture and utilization of an adaptable referral management framework to support recruitment for multiple concurrent clinical trials. The DIY-RSF can serve as a guide for leveraging common technologies to improve clinical trial recruitment procedures.

## Introduction

The ability to identify, screen, and enroll potential research participants in an efficient and timely manner is crucial to the success of clinical trials. However, researchers continually emphasize insufficient accrual and subsequent challenges [1-3], with an estimated 31% of trials failing to meet original recruitment targets and 53% of trials requiring additional time to meet study recruitment goals [4,5]. Failure to reach recruitment targets may result in a loss of statistical power and increase trial duration and costs [6,7]. Furthermore, even when studies are completed on time, a large part of the costs are associated with participant recruitment [8].

In recent years, clinical trial recruitment support systems (CTRSS) have aimed at improving the efficiency and effectiveness of clinical trial recruitment. These CTRSS use electronic patient data, typically from electronic health records or data warehouse services, to assess patient eligibility for one or more trials. The system then alerts a physician or patient of study eligibility or provides a list of potential trial participants to a study investigator [9]. Similarly, Web-based recruitment strategies have become increasingly popular, especially for electronic and mobile health (eHealth/mHealth) studies [10-12]. A 2011 systematic review concluded that prescreening is the most effective part of the recruitment process addressed by CTRSS [13]. Prescreening optimizes recruitment procedures by reserving staff time for interactions with participants who are likely to be enrolled [14]. Dividing recruitment into a series of different examinations of increasing complexity has also been shown to be successful in recruitment for large trials [15,16], although few studies describe their prescreening methods and subsequent record keeping. There are few, if any, published descriptions on optimization of these processes for eHealth clinical trials. Wide-reaching and cost-effective recruitment strategies are essential for trial success. There remains a need for systems that can function across strategies and capture data necessary to provide insight into the efficiency of different types of recruitment methods to enable investigators to appropriately allocate recruitment resources.

This paper (the first of a two-part series) describes the process of acquiring design requirements and the architecture for a practical “do-it-yourself” recruitment support framework (DIY-RSF) using technologies readily available at many academic institutions. The framework was developed to support recruitment efforts for multiple, concurrent institutional review board-approved clinical trials conducted by the Center for Behavioral Intervention Technologies (CBITs). This paper characterizes the important features of this framework and discusses the uptake and use at a single academic research center. The second part to this paper by Lattie and colleagues describes the use of the DIY-RSF to identify the most cost-effective and time-efficient recruitment strategies for eHealth clinical trials and provide a guide for recruitment decision making [17].

## Methods

### Setting

Located within the Northwestern University Feinberg School of Medicine, CBITs is an academic research center focused on developing and evaluating behavioral intervention technologies, including Web, mobile, and sensor technologies that help people make positive behavior changes to support physical and emotional health. It is supported by an interdisciplinary team of research faculty, software engineers, and research support staff. The trials at CBITs generally focus on evaluating the use and efficacy of these digital mental health interventions and tools. At the time of the needs assessment, CBITs had approximately one dozen research protocols underway, with studies requiring anywhere from five to 100 enrolled participants each month. Participant recruitment methods included digital strategies (eg, social media, Craigslist), print advertisements (eg, flyers, posters on public transportation), research registries, clinics, and traditional media strategies (eg, press releases), which directed prospective participants to the center website and/or a prescreening Web survey via various links. The second part of this paper outlines detailed descriptions of the center’s recruitment strategies [17].

### Needs Assessment

To inform the DIY-RSF, a needs assessment was conducted. A research program manager conducted a series of individual, unstructured interviews with investigators and research personnel to identify current barriers to recruitment and determine important features for the new system. Informal focus groups were also conducted during standing meetings. Interviewees were encouraged to speak freely regarding pain points with current processes and to generate a “wish list” for a new system. The research program manager then generated a prioritized list of requirements for a recruitment management framework, met with a data manager to review these requirements, and designed a general structure and workflow to support the identified needs and pain points. Some suggestions, such as interactive visualizations to show the lifecycle of a participant from the time of referral to the center through enrollment, were deemed impractical due to technology constraints. There was also a request to develop a comprehensive trial management toolkit; however, due to the magnitude of this task, it was decided early on to focus on recruitment as this was the area of greatest need in the center. System features were chosen and prioritized by determining which components of recruitment were experiencing the greatest deficiencies and the overall feasibility of implementation given the technologies available to us. Follow-up meetings were scheduled as needed to further discuss and revise the proposed DIY-RSF.

## Results

### Needs Assessment Findings

The results of the needs assessment revealed a number of challenges, inefficiencies, and wishes for a new system. Challenges included concurrent, ongoing trials with competition for participants across studies with similar goals and entry criteria, the addition of new studies over time, and a multidisciplinary research team with shared, yet unique, roles and responsibilities. Examples of overlapping entry criteria for trials included access to technology (personal computer and/or mobile phone with internet access) and at least moderate symptoms of depression and/or anxiety as measured by the Patient Health Questionnaire-9 (PHQ-9) [18] and Generalized Anxiety Disorder-7 (GAD-7) [19]. Inefficiencies included multiple disconnected systems for tracking referrals, insufficient staffing to manage time-intensive contact with the growing number of ineligible individuals, and inability for management to track recruiter caseloads in real time. Requirements for the new system are listed in Textbox 1. In developing the DIY-RSF to meet these requirements, we aimed to optimize the design to minimize participant burden and staff time.

### Recruitment Support Framework

**Figure 1:**
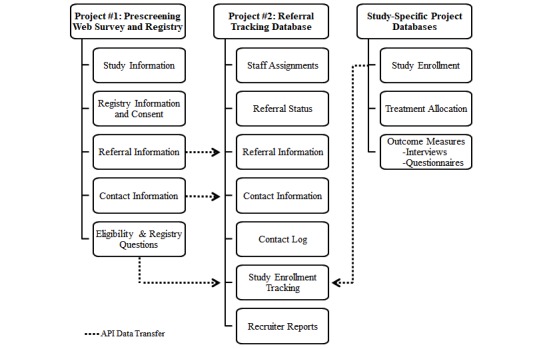
REDCap framework. API: application programming interface.

Multiple software, including REDCap (Research Electronic Data Capture); SAS software, version 9.4 (SAS Institute Inc, Cary, NC, USA); and the R programming language, version 3.4.3, supported the development and implementation of the DIY-RSF. Microsoft Excel was used to create a recruitment dashboard; however, the DIY-RSF framework does not require Excel, nor does Excel itelf support any other framework components. We deployed two different REDCap projects: (1) a prescreening Web survey and registry, and (2) a referral tracking database (Figure 1). REDCap is a Web-based platform used for collecting and managing research data in noncommercial settings [20]. Records housed in REDCap are stored to meet an institution’s local security standards. We chose the REDCap platform to create this new framework because the center already used it for traditional, study-specific data collection, our institution housed its own platform with accompanying support staff, and there were no additional technology costs to use it. We employed two separate REDCap projects to allow for new, prospective participants to take the Web survey via a public survey link and also to allow for the capture of legacy records for those who did not have prescreening data. We used SAS software and the R programming language to export data from the REDCap projects using REDCap’s application programming interface (API), perform data manipulations to make eligibility determinations, and transfer data between REDCap projects. The REDCap API is an interface that allows external programs, such as statistical software, to remotely connect to the REDCap platform and import and export data automatically via a programming script. Both SAS and R were used simply due to programmer preferences.

**Figure 2:**
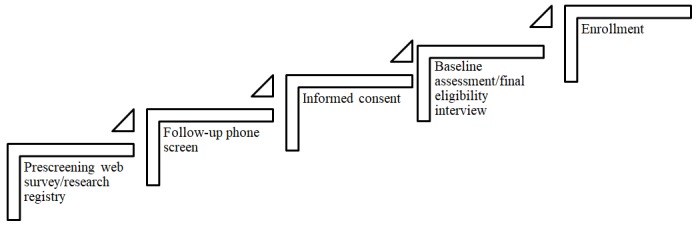
Multistage recruitment pipeline.

We modified the preexisting multistage recruitment pipeline within the center to include the prescreening Web survey, a consent form and eligibility questionnaire, a final eligibility interview, and enrollment into the trial (Figure 2). Study staff conducted a brief follow-up phone call to confirm interest and verify contact information after the prescreening Web survey for select studies. The recruitment pipeline employed a scaffold approach to screening, where the length and complexity of questions increased over time and each key point in the pipeline involved an eligibility decision. Traditional REDCap databases for housing study-specific data also played a role in the DIY-RSF via data transfer between projects. The Northwestern University Institutional Review Board (IRB) approved all registry and recruitment strategies, including all recruitment methods and materials, prescreening survey questions, informed consent language, and data handling practices.

#### REDCap Project #1: Prescreening Web Survey and Registry

The Prescreening Web Survey and Registry project is a single-survey REDCap project that also captures consent and responses to questions pertaining to a research registry. We designed this project to meet the requirements of self-referral, prescreening Web survey (requirement #1), permission of data capture on recruitment sources (requirement #3), and integration of a participant research registry (requirement #4). Prospective participants who wish to be screened for at least one CBITs clinical trial or join the center registry click the public survey link hosted on the center webpage or posted via other recruitment sources, including website and print advertisements.

First, prospective participants review a brief IRB-approved description of each study with active recruitment. After specifying study preferences, additional IRB-approved study information and/or waiver of informed consent are displayed. Participants imply consent to proceed to prescreening by continuing with the survey. Respondents then answer a series of questions regarding referral source, preliminary inclusion/exclusion criteria for studies, and general registry questions. In some instances, the referral source was prefilled in the survey by appending appropriate URL parameters to the survey link. This allowed participants to bypass the question on referral source and mitigate self-report biases. The research team made the decision to avoid asking for certain identifying or sensitive information, such as detailed mental health history, until after participants provided consent pertaining to specific study protocols.

**System requirements of the “do-it-yourself” recruitment support framework (DIY-RSF).**

1. Self-referral Web screening survey accessible via computers and mobile devices for participants
2. Ability to make automated eligibility determinations for multiple studies based on survey responses and route participants to appropriate studies
3. Ability to capture data on recruitment sources and enrollment outcomes
4. An integrated participant research registry
5. A centralized database for tracking prospective participants throughout the enrollment pipeline
6. Staff management tools that support real-time monitoring of recruiter caseloads and assignment/reassignment of cases

Prospective participants end the survey by providing contact information and preferred methods for outreach. When a participant clicks “Submit” on the last page of the survey, responses are saved in REDCap and may be accessed by the research team. This project accomplishes the need of having an efficient prescreening process that allows prospective participants to take the Web survey at any time from an internet browser or mobile device and consent to join the center research registry at the same point of contact.

#### REDCap Project #2: Referral Tracking Database

We designed the referral tracking database to serve as the central repository for data pertaining to participant recruitment sources and screening and enrollment outcomes for all trials within the center (requirement #5) and provide management with tools to support real-time monitoring of recruiter caseloads and assignment/reassignment of cases (requirement #6). The referral tracking database contains two data collection instruments: (1) referral info and (2) tracking. These data collection instruments provide an organized structure for staff to access participant data for recruitment as a whole and by study. The project is set up longitudinally with one “referral info” event and a “tracking” event for every study utilizing the DIY-RSF for recruitment. The referral tracking project serves as the “hub” for center staff to access participant records, recruiter caseloads, and basic reports for center management and staff. The referral info form contains recruiter assignments, participant referral status, detailed recruitment source information, contact information, contact preferences, and a log of all outreach attempts. The tracking form contains study-specific information concerning screening, consent, and enrollment dates, as applicable. Ultimately, this format contains eligibility determinations for each step in the recruitment pipeline and keeps a record of study enrollment across all center trials.

New referrals are batch processed daily by importing data from the prescreening survey project and reading it into SAS software via the REDCap API, which addresses the requirement to provide automated eligibility determinations for multiple studies based on survey responses and route participants to appropriate studies (requirement #2). Specifically, the API allows the REDCap and the statistical software to programmatically perform automated activities between the two systems, including reading data in directly from the referral tracking database into the SAS software without needing to manually log-in to REDCap using the user interface. A SAS program then processes survey responses to subset surveys for completed and new Web surveys since the last batch processing, merge referral tracking records by email and first and last name, and assess participant eligibility for each study based on responses to survey questions. Participants are routed to a specific study based on eligibility, study choice preferences, and center recruitment targets. The SAS program was written from scratch by the research team and includes a series of DATA steps, IF and WHERE expressions, and sorting procedures. The program code is updated as needed as studies open and close, and further upon modifications to eligibility criteria or study routing preferences. The program then generates an import template that contains a list of new referrals with their eligibility status, study to route to (if eligible), and recruitment source information. The template is formatted for the referral tracking project, which can be remotely imported to the project via the REDCap API or uploaded manually using the data import tool from within the REDCap platform.

#### Recruiter Reports

**Figure 3:**
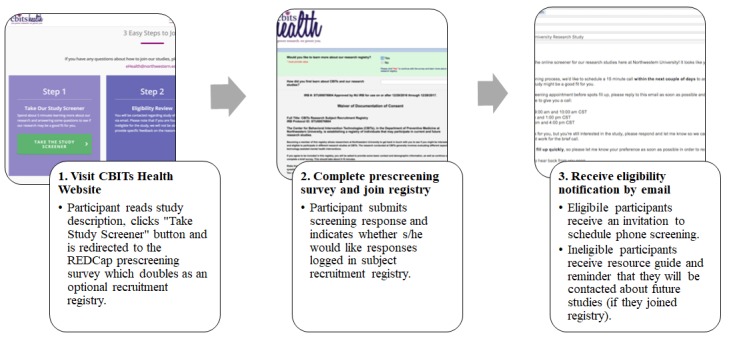
Framework structure and workflow for the participant. CBITs: Center for Behavioral Intervention Technologies.

**Figure 4:**
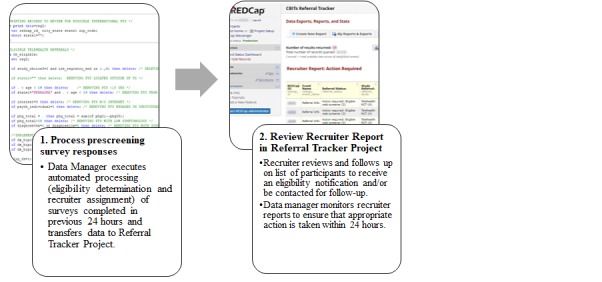
Framework structure and workflow for the data manager and recruiter.

A key component of the referral tracking project is the recruiter reports, which utilize the REDCap Data Exports, Reports, and Stats application. These are a series of individualized reports for each recruiter based on a prospective participant’s current referral status and can also be used to monitor recruiter caseloads and assign or reassign cases (requirement #6). Report filters are used to determine which records should appear on specific reports. Center workflow instructs recruiters to check reports on a daily basis and complete a series of action items, which may differ depending on a participant’s referral status. For example, the referral status of “Action required: eligible Web screener” would trigger a recruiter to attempt to contact a participant to complete the next phase of the recruitment pipeline (eg, follow-up phone screen or informed consent). A referral status of “In progress: scheduling phone screen” would advise recruiters to follow up with participants, adhering to center operating procedures regarding frequency and methods of outreach. When recruiters make appropriate updates to the participant record in the database per the action items, such as finalizing a referral status, participants will no longer appear on the reports per filter specifications. Examples of final referral status categories include “ineligible Web screener,” “could not be contacted,” or “screened.” The workflows for each stakeholder in the DIY-RSF are shown in Figures 3 and 4.

#### Recruitment Dashboard

We built an Excel dashboard to provide an at-a-glance view of key performance indicators pertaining to recruitment, such as cumulative counts of referrals and enrollment by recruitment source that can be filtered for a specified date range or trial. On a weekly basis, the REDCap API reads data into R, makes minor aggregations from data housed in the referral tracking database, and outputs an Excel template that updates the source data for the dashboard. Center staff involved in recruitment review the dashboard to maintain awareness of recruitment processes and allow for open dialog regarding reasons for lags and appropriate action, such as making adjustments to various recruitment strategies [21]. Future directions for the recruitment dashboard include improving efficiency and user interface with an R Shiny Web app [22].

### Implementation

We first launched the CBITs DIY-RSF on July 27, 2015. Prior to the launch, the center’s Data Core led a training session for all staff involved in participant recruitment. The training session utilized live demonstrations and PowerPoint, and staff received an instruction guide for continued reference after the training session. The primary cost of framework development and implementation involved staff effort. There were no outside technology costs other than those already supported at the institution and within the center (eg, SAS licenses). Development and management of the DIY-RSF were supported by a master’s-level statistician and clinical data manager and overseen by a research program manager. No new personnel were hired to support the framework and all duties pertaining to the development and management of the effort were worked into existing day-to-day operations.

#### Data Transfer

Following the initial launch, recruitment personnel were encouraged to provide feedback and suggestions for improvement about the new processes. Research assistants identified the need for manual duplicate data entry across multiple databases as a key inefficiency. In addition to compiling new records from the prescreening Web survey project and transferring to the referral tracking project, we created programming scripts using the REDCap API to transfer data between study-specific databases and the tracking form in the referral tracking database. This allowed for central access of certain information entered in the study-specific databases, such as consent and eligibility interview outcomes, from the referral tracking database (Figure 1), and it allowed center research assistants to avoid double data entry. Thus, this mitigated staff burden, improved efficiency, and reduced opportunity for human error and inconsistencies.

#### Uptake

**Figure 5:**
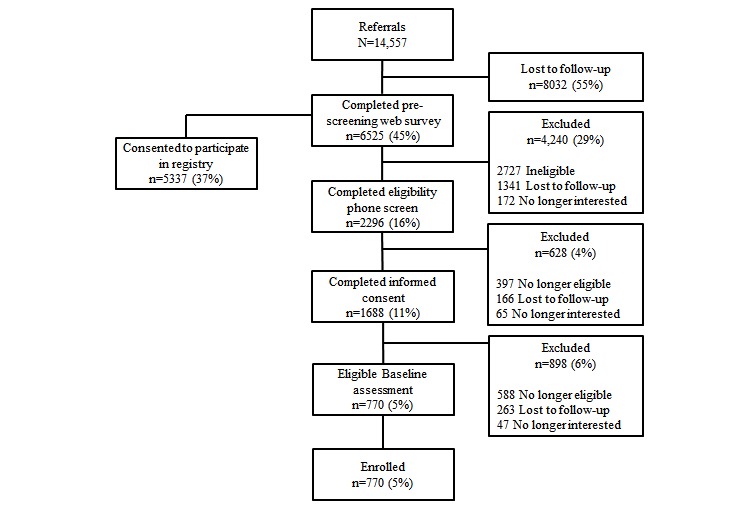
Flowchart of participant accrual outcomes at each phase of the recruitment pipeline. Note: denominator remains 14,557 referrals throughout flow diagram.

In the 2 years following the launch, the center has used the system to recruit participants for 8 research projects, including the successful completion of four NIH-funded projects, which recruited across the life span, from a project targeting youth ages 14 to 19 years, to another project targeting adults 65 years and older [23,24]. As of July 26, 2017, a total of 6525 prospective participants had completed the prescreening survey and there are currently 14,557 records entered into the referral tracking database. Of the 6525 participants who have completed the prescreening survey, 5337 (81.54%) consented to participate in the registry and agreed to be contacted at a later date for future center studies. The majority of participants enrolled reported hearing about the center via digital recruitment strategies (eg, Instagram, Reddit) and research registries (eg, ResearchMatch) [17]. Of the 14,557 records in the referral tracking database, 770 (5.29%) have been enrolled in a clinical trial. Figure 5 displays participant accrual summaries at each phase of the recruitment pipeline. Tables 1 and 2 summarize characteristics of subjects who consented to the research registry and subjects enrolled in at least one trial.

**Figure 6:**
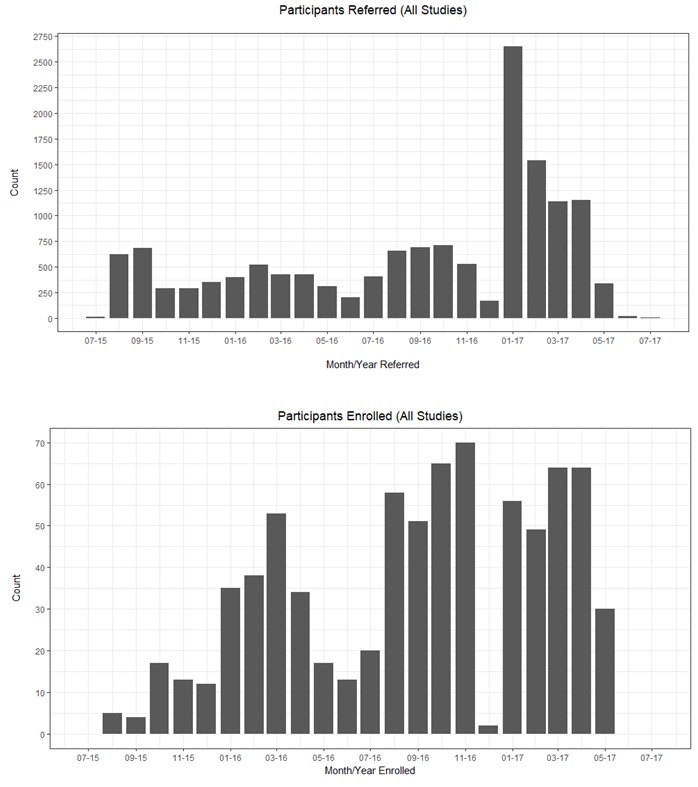
Participant referrals and enrollments over the 2 years following launch of the “do-it-yourself” recruitment support framework.

There are 453 fields, including legacy fields, across both REDCap projects. This highlights the breadth of data the framework captures. Figure 6 presents the number of referrals and enrollments in the 2-year timeframe following the launch of the DIY-RSF. In January 2017, we received a large increase in referrals due to a press release and subsequent news coverage in CBITs research. Valleys primarily correspond with holidays, such as our institution’s winter recess.

**Table 1:** Characteristics of participants in the Center for Behavioral Intervention Technologies recruitment registry and enrolled in at least one clinical trial. Percentages were calculated using nonmissing observations.

Variable		Registry participants (N=5337), n (%)	Enrolled participants (n=770), n (%)
**Gender**		**N=5333**	**N=770**
	Female	4062 (76.2)	584 (75.8)
	Male	1217 (22.8)	183 (23.8)
	Other	54 (1)	3 (0.4)
**Mobile device**		**N=5334**	**N=769**
	Yes	5141 (96.4)	741 (96.4)
	No	193 (3.6)	28 (3.6)
**Mobile device type**		**N=5136**	**N=695**
	Android	3512 (68.4)	556 (80)
	iPhone	1569 (30.5)	138 (19.9)
	Windows	27 (0.5)	0 (0)
	Other	28 (0.5)	1 (0.1)
**Access to internet**		**N=5141**	**N=770**
	Yes	5090 (99.0)	770 (100)
	No	51 (1.0)	0 (0)

**Table 2:** Characteristics of participants in the Center for Behavioral Intervention Technologies recruitment registry and enrolled in at least one clinical trial.

Variable	Registry (N=5337)		Enrolled (n=770)	
	Participants, n (%)	Mean (SD)	Participants, n (%)	Mean (SD)
Age (years)	5307 (99.44)	35.2 (13.7)	770 (100.00)	38.3 (15.6)
Patient Health Questionnaire-8^a^	5337 (100.00)	14.3 (5.4)	729 (94.68)	14.8 (4.5)
Generalized Anxiety Disorder-7	4548 (85.22)	12.2 (5.4)	578 (75.06)	12.7 (4.4)

^a^Final question omitted from Patient Health Questionnaire-9 during prescreening stage.

## Discussion

This manuscript explains the process of developing a “do-it-yourself” recruitment support framework (DIY-RSF) that streamlines and improves participant accrual for a single center in an academic setting conducting concurrent similar prospective studies. The DIY-RSF was successfully implemented in our center to support recruitment and referral management efforts. To accomplish this, we conducted a needs assessment, which identified a number of challenges and inefficiencies pertaining to current recruitment workflows. This needs assessment guided the development of our DIY-RSF. The motivation behind the implementation of the framework was to create a practical, flexible system that would mitigate current inefficiencies and support a range of trials with both overlapping and unique entry criteria and a multidisciplinary research team with varying roles and responsibilities. Since participant recruitment in clinical trials continues to be time- and cost-intensive, we used tools already supported at many academic institutions. As described previously, REDCap seemed the most logical option given the needs assessment and the availability of the platform: the local institution supported it already and made it freely available to the parties involved, which is not uncommon for those institutions housing a REDCap platform; further, REDCap is easily accessible to a wide range of skill sets with a minimal learning curve and provides an API to permit external software, such as SAS and R, to programmatically export and import data across projects.

The DIY-RSF supported automated prescreening of a large number of prospective participants and rapid identification of ineligible participants, preserving staff time for participants who have a high probability of enrolling in a trial. Other advantages include the ability to balance recruiter caseloads using features that support monitoring and assignment or reassignment of cases to other staff. The capture of objective data pertaining to study eligibility at each stage of the recruitment pipeline allowed for ongoing adjustments and optimization of recruitment strategies to improve efficiency and cost-effectiveness (see Lattie et al [17] for a more detailed description). In addition to staff-facing advantages, participant-facing advantages include the ability to take the survey from a browser or mobile device at any time and it is an easy way to send contact information. Thus, the DIY-RSF we describe in this paper is ideal for efficiently processing large numbers of prospective participants, while providing a streamlined process for participants, tools for managing staff, and data to refine recruitment processes.

The problems facing CBITs are representative of the problems facing many clinical research groups and centers as recruitment increasingly uses online modalities as the point of first contact. To date, the majority of large clinical trials within the center have been online in nature to evaluate eHealth and mHealth interventions, which did not require in-person visits with the study team. The center receives a large volume of community-based referrals with a large proportion of these referrals being ineligible or becoming lost to follow-up. “Lost to follow-up” refers to prospective participants who at one point were actively engaging in the enrollment pipeline, but were unable to be reached to move forward in the process at a point of follow-up in the recruitment process. The DIY-RSF permits a brief prescreening Web survey and automated eligibility determinations as the first step of engagement, which substantially reduces staff time spent on initial outreach and telephone screening procedures. This framework is particularly optimized for eHealth/mHealth trials that do not require in-person visits; however, many of these features may also be useful for other research groups that conduct clinic-based trials, as the internet is increasingly the medium through which people search for information, communicate, and contact research groups.

As with any new tool, the center continues to make updates to the DIY-RSF to support changing workflow of the center’s recruitment procedures, new trials, and incorporate feedback from stakeholders. Support for ongoing management and updates to the framework and associated programs are currently supported by a master’s-level data manager. We also note the center allocates a significant amount of staffing time and resources to recruitment and participant outreach, and this framework is only a single component of broader recruitment efforts within the center.

A limitation of this study is that data on referrals to the center prior to the implementation of the framework were not routinely collected and therefore prerecruitment and postrecruitment metrics are not available for comparison. The use of existing tools that were not specifically designed for recruitment processing also resulted in some limitations. First, our DIY-RSF’s capacity for data clean-up, such as reconciliation of duplicate records, was not optimal. Although the statistical program used to process referrals evaluates for duplicate records based on first and last name and email address, some instances require manual removal (eg, when survey respondents enter nicknames and secondary email addresses that the program failed to identify). Second, REDCap lacks complex visualization capabilities making visual representations of the enrollment pipeline require external software, although the API may simplify this issue. Lastly, this framework requires a strong understanding of the REDCap platform and some programming expertise for API utilization. Although the research team used SAS and R for API utilization in this framework, other languages, such as Python, PHP, Java, or Ruby, may also be used. Support is required for the ongoing management and updates to the framework and associated programs. However, we feel this time is more than offset by the more efficient use of staff time in recruitment efforts.

Using tools already supported at many academic institutions, we developed and implemented a practical referral and recruitment framework in the context of multiple concurrent trials. This framework has shown to be a valuable tool for the management and acquisition of community-based research participants in a single academic research center, particularly in regards to efficiently prescreening large volumes of participants for multiple concurrent trials. As REDCap is already supported at over 2000 institutions in over 100 countries and statistical software programs are ubiquitous in research settings, it is our hope that other research centers will be able to leverage common technologies to adapt and implement similar frameworks to support clinical trial recruitment efforts.

## Abbreviations

API: application programming interface
CBITs: Center for Behavioral Intervention Technologies
CTRSS: clinical trial recruitment support system
DIY-RSF: “do-it-yourself” recruitment support framework
IRB: institutional review board
REDCap: Research Electronic Data Capture
